# The Multidisciplinary Swallowing Team Approach Decreases Pneumonia Onset in Acute Stroke Patients

**DOI:** 10.1371/journal.pone.0154608

**Published:** 2016-05-03

**Authors:** Shiro Aoki, Naohisa Hosomi, Junko Hirayama, Masahiro Nakamori, Mineka Yoshikawa, Tomohisa Nezu, Satoshi Kubo, Yuka Nagano, Akiko Nagao, Naoya Yamane, Yuichi Nishikawa, Megumi Takamoto, Hiroki Ueno, Kazuhide Ochi, Hirofumi Maruyama, Hiromi Yamamoto, Masayasu Matsumoto, Hiroshima University Hospital Stroke Swallowing Team

**Affiliations:** 1 Department of Clinical Neuroscience and Therapeutics, Hiroshima University Graduate School of Biomedical and Health Sciences, Hiroshima, Japan; 2 Division of Nursing, Hiroshima University Hospital, Hiroshima, Japan; 3 Department of Advanced Prosthodontics, Hiroshima University Graduate School of Biomedical and Health Sciences, Hiroshima, Japan; 4 Division of Rehabilitation, Department of Clinical Practice and Support, Hiroshima University Hospital, Hiroshima, Japan; 5 Division of Nutrition Management, Hiroshima University Hospital, Hiroshima, Japan; 6 Division of Dental hygiene, Department of Clinical Practice and Support, Hiroshima University Hospital, Hiroshima, Japan; University of Münster, GERMANY

## Abstract

Dysphagia occurs in acute stroke patients at high rates, and many of them develop aspiration pneumonia. Team approaches with the cooperation of various professionals have the power to improve the quality of medical care, utilizing the specialized knowledge and skills of each professional. In our hospital, a multidisciplinary participatory swallowing team was organized. The aim of this study was to clarify the influence of a team approach on dysphagia by comparing the rates of pneumonia in acute stroke patients prior to and post team organization. All consecutive acute stroke patients who were admitted to our hospital between April 2009 and March 2014 were registered. We analyzed the difference in the rate of pneumonia onset between the periods before team organization (prior period) and after team organization (post period). Univariate and multivariate analyses were performed using a Cox proportional hazards model to determine the predictors of pneumonia. We recruited 132 acute stroke patients from the prior period and 173 patients from the post period. Pneumonia onset was less frequent in the post period compared with the prior period (6.9% vs. 15.9%, respectively; p = 0.01). Based on a multivariate analysis using a Cox proportional hazards model, it was determined that a swallowing team approach was related to pneumonia onset independent from the National Institutes of Health Stroke Scale score on admission (adjusted hazard ratio 0.41, 95% confidence interval 0.19–0.84, p = 0.02). The multidisciplinary participatory swallowing team effectively decreased the pneumonia onset in acute stroke patients.

## Introduction

Dysphagia occurs in acute stroke patients at high rates [1, 2], and many of these patients develop aspiration pneumonia [3, 4]. Pneumonia incurs extended hospitalization and decreases the rate of hospital discharge [5]. Therefore, the early intervention in dysphagia is important to prevent aspiration pneumonia in acute stroke patients. Recently, major two clinical trials were reported to assess the effectiveness of antibiotic prophylaxis for reducing pneumonia in acute stroke patients [6, 7]. Unfortunately, antibiotic prophylaxis cannot effectively prevent of post-stroke pneumonia in both two studies. It is now uncertain what way is the effective to reduce pneumonia in acute stroke patients.

Team approaches with the cooperation of various professionals have the power to improving the quality of medical care, utilizing the specialized knowledge and skills of each profession [8]. Thus, we consider that multidisciplinary team approaches have the potential for reducing pneumonia onset in acute stroke patients. However, few studies demonstrate that the multidisciplinary participatory swallowing team approach significantly decreases the rate of pneumonia in acute stroke patients. In our hospital, a multidisciplinary participatory swallowing team was organized in April 2011. The aim of this study was to clarify the influence of the team approach on dysphagia by comparing the rates of pneumonia in acute stroke patients between the prior and post team periods.

## Materials and Methods

All consecutive acute stroke patients who were admitted to our hospital between April 2009 and March 2014 were registered. A multidisciplinary participatory swallowing team was organized in April 2011. Thus, we defined the period before team organization (April 2009 to March 2011) as the ‘prior period’ and the period after team organization (April 2011 to March 2014) as the ‘post period’. The patients in the prior period were analyzed as a historical control. The multidisciplinary participatory swallowing team in our hospital consists of 9 professionals, including doctors, dentists, nurses, physical therapists, occupational therapists, speech therapists, managerial dieticians, dental hygienists, and pharmacists.

Two neurologists reviewed the charts of all consecutive acute stroke patients blind the period of admission. Clinical parameters (age, sex, vascular risk factors [hypertension, diabetes mellitus, and dyslipidemia], smoking, previous stroke, stroke severity, and stroke subtype), body temperature, laboratory findings (C-reactive protein [CRP] and white blood cell [WBC] count), and radiological findings (chest X-ray, computed tomography [CT], and magnetic resonance imaging [MRI]) were recorded for all patients during hospitalization. Hypertension was defined as the use of anti-hypertensive medicines prior to admission or a confirmed blood pressure ≥140/90 mmHg at rest 2 weeks after stroke onset. Diabetes mellitus was defined as an HbA1c of ≥6.5%, a fasting blood sugar ≥126 mg/dl, or the use of anti-diabetic medicines. Hyperlipidemia was defined as a total cholesterol ≥220 mg/dl, a low-density lipoprotein cholesterol ≥140 mg/dl at admission, or the use of anti-hyperlipidemia medications. Smoking was defined according to the definition of the US Centers for Disease Control and Prevention [9] as follows: (1) never smokers, who had never smoked a cigarette or who smoked fewer than 100 cigarettes in their entire lifetime; (2) former smokers, who had smoked at least 100 cigarettes in their lifetime, but said they currently did not smoke; and (3) current smokers, who have smoked 100 cigarettes in their lifetime and currently smoke cigarettes every day (daily) or some days (nondaily).

The diagnosis of clinically defined pneumonia was based on the criteria of the Centers for Disease Control and Prevention [10] as follows: Clinically defined pneumonia criteria require the presence of a new and persistent infiltrate or consolidation on at least 1 chest X-ray or CT with one of the following clinical signs: fever, leukopenia or leukocytosis and altered mental status in more than 70-year-olds in the absence of other causes. These should be added to 2 of the following signs: new-onset purulent sputum or change in the character of the sputum, new-onset or progressive cough, rales, and impaired gas exchange. We evaluated the difference in the rate of pneumonia onset between the prior and post team organization periods as well as the factors that influenced pneumonia onset.

All patients underwent head CT or MRI. The stroke subtype was determined based on the CT or MRI findings, electrocardiography, and carotid artery and cardiac ultrasound findings by at least two stroke specialists according to the Trial of Org 10172 in Acute Stroke Treatment (TOAST) classification [11]. The neurological severity was evaluated using the National Institutes of Health Stroke Scale (NIHSS) score at admission [12]. This study was approved by the Institutional Review Board (IRB) of Hiroshima university hospital (E-144). All clinical investigation must have been conducted according to the principles expressed in the Declaration of Helsinki. Because the data were analyzed anonymously, no informed consent was given.

Statistical analyses were performed using the JMP 12.0.1 statistical software (SAS Institute, Inc., Cary, North Carolina, USA). The data are presented as the mean ± standard deviation (SD) or the median (minimum–maximum) for continuous variables. Statistical analyses of the comparisons of the two groups were performed using Student’s t-test or the Mann-Whitney U test for continuous variables and the chi-square test or Fisher’s exact test for categorical variables. Two tail p-values <0.05 were considered statistically significant. Univariate and multivariate analyses were performed using a Cox proportional-hazards model to determine the predictors of pneumonia. The Cox proportional hazard model was used to estimate the relative risk (hazard ratio, HR) and the 95% confidence interval (CI). The cumulative incidences of time to the onset of pneumonia were estimated using the Kaplan-Meier method. The cumulative incidence curves for the two groups (patients in the prior period vs. patients in the post period) were compared using a log-rank test.

## Results

**Fig 1:**
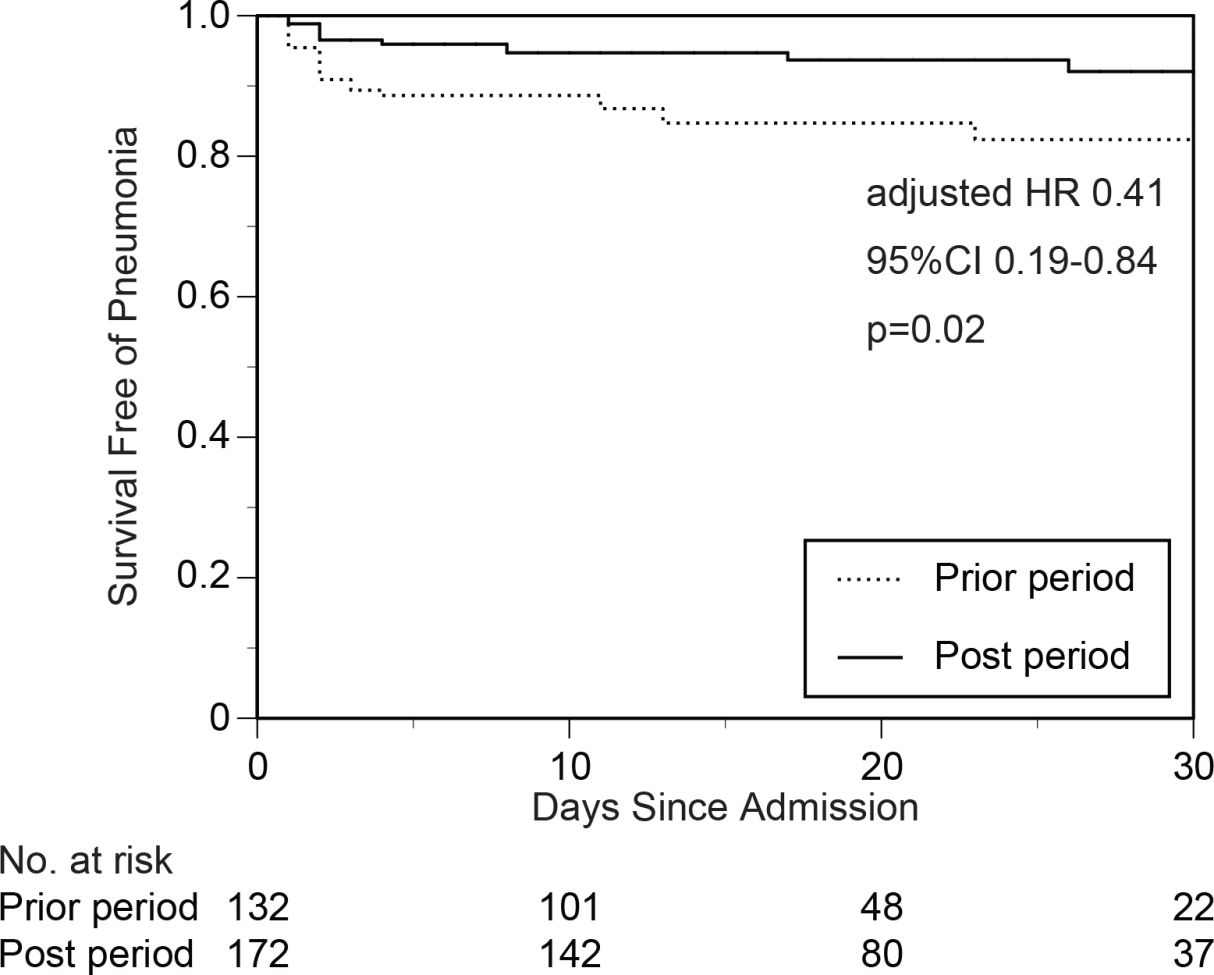
Probability of not developing pneumonia in the prior period and in the post period. Pneumonia onset was less frequent in the post period compared with the prior period.

We recruited 132 acute stroke patients from the prior period (April 2009 to March 2011) and 173 patients from the post period (April 2011 to March 2014). Age, sex, vascular risk factors, NIHSS score on admission, and stroke subtype did not significantly differ between the two groups (Table 1). No patients received prophylactic antibiotics. The rates of patients having a fever with temperatures greater than 38°C did not significantly differ between the two groups, but the rates of patients with an increasing WBC count and CRP were significantly reduced in the post period compared with the prior period. Pneumonia onset was less frequent in the post period compared with the prior period (6.9% vs. 15.9%, respectively; p = 0.01, Table 2, Fig 1). Using an univariate analysis with a Cox proportional-hazards model, it was determined that NIHSS score on admission and the application of a swallowing team approach were significantly related to pneumonia onset (Table 3). Using a multivariate analysis with the factors selected in univariate analyses as statistically significant, it was determined that NIHSS score on admission and the application of a swallowing team approach were independently related to pneumonia onset (Table 4). To understand the difference of swallowing dependent approaches between the prior and post period, frequencies of professional oral care and swallowing evaluations (videoendoscopic examination of swallowing [VE] or videofluoroscopic examination of swallowing [VF]) were evaluated (Table 5). The rates of patients receiving professional oral care and swallowing evaluations were significantly increased in the post period compared with the prior period.

**Table 1:** Baseline Characteristics of the Patients.

Factors	Patients in the prior period (n = 132)	Patients in the post period (n = 173)	p-value
Age, years	70.0±12.2	70.1±11.5	0.91
Sex, female	46 (34.9%)	63 (36.4%)	0.78
Vascular risk factors			
Hypertension	88 (66.7%)	130 (75.1%)	0.11
Diabetes mellitus	51 (38.6%)	51 (29.5%)	0.09
Dyslipidemia	45 (34.1%)	72 (41.6%)	0.18
Smoking			0.82
Never smokers	66 (50.0%)	85 (49.1%)	
Former smokers	38 (28.8%)	55 (31.8%)	
Current smokers	28 (21.2%)	33 (19.1%)	
Previous stroke	32 (24.2%)	41 (23.7%)	0.91
NIHSS score at admission, median (IQR)	5 (2–13)	5 (2–14)	0.60
Stroke subtype			0.62
Cardioembolism	41 (31.1%)	51 (29.5%)	
Large artery atherosclerosis	22 (16.6%)	25 (14.5%)	
Small artery occlusion	12 (9.1%)	18 (10.4%)	
Other mechanisms	29 (22.0%)	36 (20.8%)	
Hemorrhage	28 (21.2%)	43 (24.8%)	

NIHSS, the National Institutes of Health Stroke Scale; IQR, interquartile range.

**Table 2:** Findings During Hospitalization.

Factors	Patients in the prior period (n = 132)	Patients in the post period (n = 173)	p-value
Fever (≥38.0°C)	29 (22.0%)	36 (20.8%)	0.81
WBC (≥10,000/μl)	38 (28.8%)	32 (18.5%)	0.03
CRP (≥2.0 mg/dl)	50 (37.9%)	44 (25.4%)	0.02
Pneumonia	21 (15.9%)	12 (6.9%)	0.01

WBC, white blood cell; CRP, C-reactive protein.

**Table 3:** Univariate Analyses Using a Cox Proportional Hazards Model to Determine Associations with Pneumonia.

Factors	Hazard ratio	95% confidence interval	p-value
Age (per increase 1 year)	1.02	0.99–1.05	0.21
Male	1.22	0.59–2.70	0.61
Hypertension	0.97	0.46–2.23	0.94
Diabetes mellitus	0.46	0.17–1.05	0.07
Dyslipidemia	0.57	0.24–1.23	0.16
Previous stroke	1.25	0.55–3.37	0.61
NIHSS on admission (per increase 1 point)	1.11	1.08–1.14	<0.0001
Swallowing team approach	0.39	0.18–0.81	0.01

NIHSS, the National Institutes of Health Stroke Scale.

**Table 4:** Multivariate Analyses Using a Cox Proportional Hazards Model to Determine Associations with Pneumonia.

Factors	Hazard ratio	95% confidence interval	p-value
NIHSS on admission (per increase 1 point)	1.11	1.07–1.14	<0.0001
Swallowing team approach	0.41	0.19–0.84	0.02

**Table 5:** Patients Received Professional Oral Care and Swallowing Evaluations (VE or VF).

Factors	Patients in the prior period (n = 132)	Patients in the post period (n = 173)	p-value
Professional oral care	17 (12.9%)	90 (51.7%)	<0.0001
Swallowing evaluations	16 (12.1%)	45 (26.0%)	0.002

VE, videoendoscopic examination of swallowing; VF, videofluoroscopic examination of swallowing.

## Discussion

In this study, we demonstrated that the multidisciplinary participatory swallowing team approach effectively decreases the onset of pneumonia in acute stroke patients. Pneumonia after stroke has been implicated in morbidity, mortality, and increased medical costs after acute stroke [4, 5]. Thus, the prevention of pneumonia after an acute stroke is of great importance.

Several previous studies reported that stroke severity on admission [13, 14], dysphagia [15, 16], and old age [17, 18] are major independent risk factors for the onset of pneumonia after stroke. In our study, NIHSS score on admission and the application of a swallowing team approach were independently related to the onset of pneumonia. The factor of stroke severity on admission is common to both previous studies and our study. Old age was not related to the pneumonia in our study. No studies demonstrate that the multidisciplinary participatory swallowing team approach significantly decreases the rate of pneumonia in acute stroke patients. Therefore, we believe that our results provide great value in improving acute stroke management.

There are several possibilities why the multidisciplinary participatory swallowing team approach significantly decreases the rate of pneumonia. The first reason is that an increasing number of patients received professional oral care by dentists or dental hygienists. In the period before the team organization, nurses performed routine oral care to all patients, but the rate of patients receiving professional oral care was only 12.9%. However, the rate of patients receiving professional oral care was raised up to 51.7% in the period after team organization. Several previous studies demonstrated that professional oral care appears to reduce the incidence of pneumonia in elderly individuals but not in acute stroke patients [19, 20]. The second reason is that an increasing number of patients received a swallowing evaluation (VE or VF). The rate of patients receiving a swallowing evaluation was 12.1% in the period before the team was organized but 26.0% in the period after the team was organized. The third reason is that we were able to provide managerial dieticians who created appropriate dysphagia diets and nutritional supplements based on the general condition and neurological prognosis of the patients. One report suggests that under-nutrition is independently related to post-stroke complications (including pneumonia) and clinical outcomes [21]. Therefore, an improvement in nutritional management may decrease the rate of pneumonia. The fourth reason is that physical therapists and occupational therapists improved patient body positions at mealtimes. Acceptable body positions at mealtimes are important to prevent aspiration. We think that an improvement in body position may also contribute to decreasing of pneumonia.

Additionally, by organizing the multidisciplinary participatory swallowing team, each profession could communicate very smoothly and share the detailed information of all stroke patients early in the admission. We consider that this face-to-face communication led the intervention of each profession with proper timing and contributed to decreasing pneumonia onset. Before the team organization, the needs to intervene of each profession were decided by only the attending doctors’ assessments. However, the multidisciplinary participatory swallowing team offered a variety of perspectives on the patients and resulted in the improvement of the quality of acute stroke care. All patients before team organization were received oral care by floor nurses including the certificated dysphagia nurse. The attending doctors provided swallowing evaluation of each patient by modified water swallow test at bedside, and decided the food form. When the attending doctors estimated that the patient was needed to receive professional oral care or swallowing evaluations (videoendoscopic examination of swallowing [VE] or videofluoroscopic examination of swallowing [VF]), dentists and speech therapists intervened in the patients. All patients were received general acute stroke rehabilitation by physical therapists and occupational therapists. Basically, the acute treatment and other general medical care were determined based on the stroke subtype in accordance with the same established guideline (the Japanese Guidelines for the Management of Stroke 2009) both in historical control and study cohorts. The practices from each profession were not different between the prior and post period, although proportions of patients who got professional oral care or swallowing evaluations were substantially higher in the post period. Their increases may come from face-to-face professional communications.

Various previous studies reported that pneumonia during hospitalization in acute stroke patients is related to an increasing length of hospital stay [17, 18, 22]. In our study, the length of hospital stay was significantly longer in patients with pneumonia compared with patients without pneumonia (36.5±22.2 days vs. 20.2±11.6 days, p<0.0001). However, the length of hospital stay did not significantly differ between patients in the prior period and patients in the post period. We attribute this lack of difference to the fact that the rate of patients with pneumonia was relatively low in both groups. Thus, the influence of pneumonia on the length of hospital stay was limited.

Some medications are known to prevent aspiration pneumonia. Angiotensin-converting enzyme inhibitors [23] and cilostazol [24] were reported to reduce aspiration pneumonia in stroke patients. In our study, the rates of patients taking these medications were similar (13.6% in the prior period, 15.0% in the post period). Therefore, we consider that the effectiveness of these medications were limited in our study.

There are some limitations to our study. First, this is a single-center study that evaluates the influence of the multidisciplinary participatory swallowing team approach on the onset of pneumonia compared with a historical control. Therefore, we cannot exclude the possibility that a selection bias exists. However, it is difficult to conduct a randomized study of the multidisciplinary participatory swallowing team approach. Second, there are few objective indicators of the effects of the multidisciplinary participatory swallowing team approach in this study. To evaluate the effects of the swallowing team approach more objectively, we need quantitative indicators, such as bacterial counts in the oral cavity that reflect the effect of oral care and serum albumin levels that reflect the effect of nutrition management.

## Conclusions

Our study demonstrates that the multidisciplinary participatory swallowing team approach effectively decreases the onset of pneumonia in acute stroke patients. Further prospective multicenter studies using more objective indicators are necessary to clarify the effect of the multidisciplinary participatory swallowing team approach.

## Supporting Information

S1 File The members of the Hiroshima University Hospital Stroke Swallowing Team. (PDF)
