# Expression of Concern: The Multidisciplinary Swallowing Team Approach Decreases Pneumonia Onset in Acute Stroke Patients

**DOI:** 10.1371/journal.pone.0279798

**Published:** 2023-01-11

**Authors:** The PLOS ONE Editors

The Funding Statement for this article [1] states that the study received funding from the Smoking Research Foundation, which according to [2] has received financial support from the tobacco industry. In light of this issue the *PLOS ONE* article [1] does not comply with the journal’s policy on Funding from Tobacco Companies [3] which was implemented in 2010. Therefore, the *PLOS ONE* Editors issue this Expression of Concern.

We regret that this concern was not identified and addressed prior to the article’s [1] publication.

## References

[1] Aoki S, Hosomi N, Hirayama J, Nakamori M, Yoshikawa M, Nezu T, et al. (2016) The Multidisciplinary Swallowing Team Approach Decreases Pneumonia Onset in Acute Stroke Patients. PLoS ONE 11(5): e0154608. 10.1371/journal.pone.0154608 27138162 PMC4854465

[2] Iida K, Proctor RN. (2018) ‘The industry must be inconspicuous’: Japan Tobacco’s corruption of science and health policy via the Smoking Research Foundation. Tobacco Control 27:e3–e11. doi: 10.1136/tobaccocontrol-2017-053971 29437992 PMC6073917

[3] https://journals.plos.org/plosone/s/disclosure-of-funding-sources#loc-funding-from-tobacco-companies

