# Supplementary material for: The Multidisciplinary Swallowing Team Approach Decreases Pneumonia Onset in Acute Stroke Patients
Source: PLoS One. 2016 May 3;11(5):e0154608. doi: 10.1371/journal.pone.0154608 (PMC4854465; doi:10.1371/journal.pone.0154608)
Supplement: S1 File — (PDF) [file pone.0154608.s001.pdf]

S1: Hiroshima University Hospital Stroke Swallowing Team

Doctors

Shiro Aoki, Mutsuko Araki, Eisuke Dohi, Hiroki Fujii, Shotaro Haji, Naohisa Hosomi, Naoto Kinoshita, Juri Kitamura, Megumi Kobayashi, Michihiro Kono, Tomoyuki Kono, Teppei Kotozaki, Satoshi Kubo, Yuta Maetani, Hirofumi Maruyama, Masayasu Matsumoto, Hayato Matsushima, Yoshito Nagano, Hiroyuki Naito, Masahiro Nakamori, Takeshi Nakamura, Tomohisa Nezu, Nishikawa Tomokazu, Kazuhide Ochi, Yoko Ota, Yukari Shinozaki, Takeo Shishido, Tomohisa Sugiura, Hiroki Ueno, Takeshi Yoshimoto, Yuu Yamazaki

Dentists

Chiaki Higa, Aya Hiraoka, Takahiro Mori, Hiromi Nishi, Mineka Yoshikawa

Nurses

Ayame Abe, Hiroko Amano, Ayako Azechi, Ayaka Fukumoto, Kotomi Hamada,

Natsuyo Hamasaki, Miu Higawa, Junko Hirayama, Mina Ishihara, Kozue Kagotani, Saya Kamikawa, Shingo Kishita, Makiko Kiyohara, Kiyomi Kobayashi, Mika Kogawa, Keiko Madokoro, Masao Maeda, Miyuki Maeoki, Yoshiko Masuda, Yumi Matsuo, Haruka Miyake, Rie Miyamoto, Teruyo Moriyama, , Yasuko Motoyama, Miki Nishiyama, Kahori Nuruyu, Takashi Okada, Nanako Oyama, Masakazu Ryuji, Natsuko Sako, Aya Sasaki, Tomomi Sasaki, Yoko Sato, Takae Seki, Haruka Shimizu, Yumi Takahara, Megumi Tanaka, Miki Tanaka, Isamu Tominaga, Shouko Tomioka, Sayaka Yamaichi, Hiromi Yamamoto, Kasumi Yanagihara

Physical therapists

Yuichi Nishikawa, Naoya Orita, Noboru Shimada

Occupational therapists

Naoya Yamane

Speech therapists

Yuka Nagano, Mariko Ueasa, Akiko Yoshimura

Managerial dieticians

Kanako Amano, Akiko Nagao, Miyuki Oohata, Kazumi Shimada, Hiroko Tabuchi

Dental hygienists

Tomomi Okino, Megumi Takamoto, Kotomi Tetsumori
